# Editorial: Clinical trial design and development in neonatal and perinatal medicine

**DOI:** 10.3389/fped.2025.1557059

**Published:** 2025-02-06

**Authors:** Mohamed E. Abdel-Latif, Yogavijayan Kandasamy, Sabita Uthaya, Dirk Bassler, Jonathan M. Davis

**Affiliations:** ^1^ Department of Neonatology, Centenary Hospital for Women and Children, Canberra Hospital, Canberra, ACT, Australia; ^2^ Discipline of Neonatology, School of Medicine and Psychology, College of Health and Medicine, Australian National University, Canberra, ACT, Australia; ^3^ Department of Public Health, College of Science Health and Engineering, La Trobe University, Melbourne, VIC, Australia; ^4^ Department of Neonatology, Townsville University Hospital, Townsville, QLD, Australia; ^5^ College of Medicine and Dentistry, James Cook University, Townsville, QLD, Australia; ^6^ Conjoint Research Fellow, School of Medicine and Public Health, The University of Newcastle, Newcastle, NSW, Australia; ^7^ Neonatal Medicine, Department of Primary Care and Public Health, Imperial College London, London, United Kingdom; ^8^ Neonatal Services, Chelsea and Westminster Hospital, London, United Kingdom; ^9^ Department of Neonatology, University of Zurich, Zurich, Switzerland; ^10^ Department of Neonatology, University Hospital Zurich, Zurich, Switzerland; ^11^ Department of Pediatrics, Tufts Medical Center, Boston, MA, United States; ^12^ Pediatrics and Newborn Medicine, Tufts Clinical and Translational Science Institute, Boston, MA, United States

**Keywords:** trial, neonate, perinatal, design, studies

**Editorial on the Research Topic**
Clinical trial design and development in neonatal and perinatal medicine

Recent evidence from perinatal trials has significantly improved neonatal care, leading to a reduction in neonatal mortality, morbidity, and long-term disability. These advancements have integrated clinical trials into neonatal and perinatal practice and facilitated progress in the expanding Clinical Trial Design and Development field in Neonatal and Perinatal Medicine. Frontiers in Pediatrics has organized a series of Research Topics focused on clinical trials in Neonatal and Perinatal Medicine to highlight the latest advancements in this field. This editorial addresses new insights, novel developments, ongoing challenges, recent discoveries, and future perspectives in the field. This Research Topic aims to illuminate key advancements achieved over the past decade in Clinical Trials related to Neonatal and Perinatal Medicine. This Special Issue seeks to bridge the gap between research and clinical practice, fostering a deeper understanding of new possibilities and encouraging discussions within the perinatal and neonatal medicine communities. The thematic series covers various topics of interest to the perinatal and neonatal communities.

In the fields of pharmacokinetics and pharmacodynamics, Yeung et al. highlighted the principles and unique considerations for optimal design of neonatal clinical trials. Jilani et al., through a literature review applying the RAND/UCLA Appropriateness Method and thematic analysis, found that the interactional relationship between the opioid-exposed birthing person and the infant is the foundational principle that clinically defines the dyad to better support bedside care, surveillance, and research. Jumani et al. conducted a multicenter retrospective study to evaluate the short-term effects of opioids during therapeutic hypothermia (TH) for neonatal encephalopathy. Their study showed that opioid use during TH was associated with adverse short-term outcomes and highlighted the need for longer-term cohort studies. Köber et al. found that the gestational age at birth, birth weight, and gestational age at the time of intrauterine brain sparing determine neonatal outcomes in growth-restricted infants born before 32 weeks of gestation. Furthermore, a small subset of data showed that the nitric oxide donor pentaerythrityl tetranitrate (PETN) may ameliorate the effect of brain sparing in the affected neonates. The work of Pei and Chen explored the association between dexamethasone usage prior to elective full-term cesarean delivery and short-term adverse neonatal outcomes using a retrospective cohort study design. Their results suggest the importance of exercising caution when contemplating the use of antenatal corticosteroids-prior to elective full-term cesarean delivery. Allegaert et al. reported on the current status, trajectory, stakeholder assessment, impact, and future perspectives of the Neonatal Adverse Event Severity Scale (NAESS). This tool was developed by the International Neonatal Consortium (INC) to standardize assessment, facilitate reproducibility of results, and assess the severity and causality of adverse events in clinical trials.

Finally, Degl et al. provided the perspective of two parents of premature infants and two neonatal nurses on the partnership and strong bond that develops between parents and NICU nurses. With nurses being a constant calming presence at the bedside, a trusting bond between parents and nurses often helps to promote clinical research and becomes the lifeline to survive the NICU journey. Fully realizing this “bond” will improve outcomes for patients and families and contribute to the growth and success of the entire NICU system.

In conclusion, we hope that this collection of articles, which showcases a variety of complementary contributions from various institutions worldwide, will inform, guide, and inspire researchers in the field of perinatal and neonatal medicine and continue to lead to improvements in survival and outcomes.

